# A Novel Type of Neuron Within the Dorsal Striatum

**DOI:** 10.3389/fncir.2019.00032

**Published:** 2019-05-21

**Authors:** Miaomiao Mao, Aditya Nair, George J. Augustine

**Affiliations:** ^1^Lee Kong Chian School of Medicine, Nanyang Technological University, Singapore, Singapore; ^2^Institute of Molecular and Cell Biology, Singapore, Singapore; ^3^Life Sciences Programme, Faculty of Science, National University of Singapore, Singapore, Singapore; ^4^Singapore Bioimaging Consortium, Agency of Science, Technology and Research, Singapore, Singapore

**Keywords:** interneurons, neuron diversity, dorsal striatum, tyrosine hydroxylase, intrinsic electrical properties, neuron classification

## Abstract

The striatum is predominantly composed of medium spiny projection neurons, with the remaining neurons consisting of several types of interneurons. Among the interneurons are a group of cells that express tyrosine hydroxylase (TH). Although the intrinsic electrical properties of these TH-expressing interneurons have been characterized, there is no agreement on the number of TH-expressing cell types and their electrical properties. Here, we have used transgenic mice in which YFP-tagged channelrhodopsin-2 (ChR2) was expressed in potential TH-expressing cells in a Cre-dependent manner. We found that the YFP^+^ neurons in the striatum were heterogeneous in their intrinsic electrical properties; unbiased clustering indicated that there are three main neuronal subtypes. One population of neurons had aspiny dendrites with high-frequency action potential (AP) firing and plateau potentials, resembling the TH interneurons (THIN) described previously. A second, very small population of labeled neurons resembled medium-sized spiny neurons (MSN). The third population of neurons had dendrites with an intermediate density of spines, showed substantial AP adaptation and generated prolonged spikes. This type of striatal neuron has not been previously identified in the adult mouse and we have named it the Frequency-Adapting Neuron with Spines (FANS). Because of their distinctive properties, FANS may play a unique role in striatal information processing.

## Introduction

The striatum is a major component of the basal ganglia circuit and is involved in a number of functions such as motor planning, motivation and reward perception. Medium-sized spiny neurons (MSN) are the most abundant cell type in the striatum and constitute about 95% of all striatal neurons in rodents (Graveland and DiFiglia, 1985; Kreitzer, 2009). MSN are GABAergic and are the only known projection neurons of the striatum. The remaining striatal neurons are made up of several kinds of interneurons that differ in their neurochemical and electrophysiological characteristics: (1) fast-spiking (FS) parvalbumin (PV)-expressing GABAergic interneurons; (2) low-threshold Ca^2+^ spiking (LTS) somatostatin (SOM)/neuropeptide Y (NPY)/nitric oxide synthase (NOS)-expressing GABAergic interneurons; and (3) spontaneously active cholinergic interneurons. In addition, calretinin-expressing GABAergic interneurons have also been observed in immunohistochemical studies but their electrophysiological properties are unknown (Rymar et al., 2004; Tepper et al., 2010). More recently, the use of transgenic mice expressing neuron-specific markers has revealed still other types of striatal interneurons. These include a NPY-expressing interneuron that differs from the NPY-LTS cell (Ibáñez-Sandoval et al., 2011; Assous et al., 2017), as well as a heterogeneous population of interneurons that express 5HT3a receptors (Faust et al., 2015, 2016; Muñoz-Manchado et al., 2016).

An additional population of tyrosine hydroxylase (TH)-immunoreactive (TH^+^) interneurons has been found in the striatum of several species, including primates (Dubach et al., 1987), rat (Tashiro et al., 1989), mouse (Mao et al., 2001) and human (Ikemoto et al., 1997; Cossette et al., 2005). These neurons are up-regulated after denervation of dopaminergic input from the midbrain (Nakahara et al., 2001; Tandé et al., 2006; Darmopil et al., 2008) and their morphology, electrophysiological properties and distribution have been characterized (Ibáñez-Sandoval et al., 2010; Masuda et al., 2011; Ünal et al., 2011). These characterizations have yielded somewhat inconsistent conclusions; specifically, it is currently unclear whether there are two (Masuda et al., 2011) or four (Ibáñez-Sandoval et al., 2010) subtypes of TH-expressing neurons.

Here, we have extended the genetic approach to characterize striatal neurons in transgenic mice in which YFP-tagged Channelrhodopsin-2 (ChR2) expression was driven by Cre expression in a TH-Cre mouse line. We found that YFP^+^ striatal neurons were quite heterogeneous in their intrinsic electrical properties and could be sorted, by an unsupervised clustering method, into three main groups. More than 20% of the neurons resembled the TH-expressing interneurons (THIN) described previously (Ibáñez-Sandoval et al., 2010), while a small fraction of neurons closely resembled typical MSN. A third group of cells had unique electrophysiological properties that did not resemble those of THIN, MSN, or any other striatal cell that has previously been described. These cells showed substantial action potential (AP) adaptation, generated prolonged spikes in response to depolarizing currents and possessed dendrites with an intermediate density of spines. We have named these novel neurons Frequency-Adapting Neurons with Spines (FANS) and here we compare the properties of FANS to those of the other YFP^+^ cells within the dorsal striatum in our transgenic mice.

## Materials and Methods

### Animals

Transgenic mice expressing ChR2 fused to YFP in TH-Cre expressing neurons were generated by mating a line of TH-Cre transgenic mice (Lindeberg et al., 2004) with another line (Ai32) expressing YFP-tagged ChR2 behind a floxed stop cassette (Madisen et al., 2012). Both mouse lines were obtained from Jackson Labs. All mice used in the experiments were of either sex and age 6–14 weeks; all animal procedures were approved by the Institutional Animal Care and Use Committee of Biopolis.

### Preparation of Brain Slices

Mice were anesthetized with isoflurane and euthanized *via* decapitation. Brains were removed quickly and placed in carbogen-saturated ice-cold cutting solution containing the following (in mM): 250 sucrose, 26 NaHCO_3_, 10 glucose, 2.5 KCl, 1.25 HaH_2_PO_4_·H2O, 3 myoinositol, 2 sodium pyruvate, 4 MgCl_2_, 0.1 CaCl_2_, 0.5 ascorbic acid and 1 kynurenic acid. 300 μm thick parasagittal slices containing the dorsal striatum were cut with a vibratome and then transferred to a recovery chamber containing carbogen-saturated artificial cerebrospinal fluid (ACSF) of the following composition (in mM): 126 NaCl, 24 NaHCO_3_, 10 glucose, 2.5 KCl, 1 HaH_2_PO_4_·H2O, 2 MgCl_2_, 2 CaCl_2_, 0.4 ascorbic acid. The brain slices were incubated at 34°C for 1 h before being returned to room temperature for recording in ACSF.

### Electrophysiology

ChR2-YFP expressing cells in the dorsal striatum were visualized using a 25× objective (NA = 1.05) on an Olympus FV-1000 two-photon microscope. Neurons expressing ChR2-YFP were activated by blue light (BP470-495 excitation filter, Olympus) from a Mercury arc lamp. The duration of the light flashes was controlled by an electronic shutter (Uniblitz Model T132, Vincent Associates). Whole-cell patch clamp recordings were made using a MultiClamp 700B amplifier (Molecular Devices) and were performed under visual guidance. Recording pipettes (6–8 MΩ resistance) were filled with an internal solution containing (in mM, unless otherwise stated): 130 potassium gluconate, 10 KOH, 2.5 MgCl_2_, 10 HEPES, 4 Na_2_ATP, 0.4 Na_3_GTP, 5 EGTA, 5 disodium phosphocreatine, 0.025 Alexa fluor 594 (Invitrogen) and 0.2% (w/v) neurobiotin (Vector Labs). All reagents were obtained from Sigma-Aldrich unless otherwise indicated. Series resistance and whole-cell compensation were performed immediately after establishing the whole-cell recording configuration, while bridge balance was performed in current-clamp mode. Recordings with series resistance greater than 25 MΩ, resting membrane potential (RMP) more depolarized than −50 mV or fluctuations greater than 10% were excluded from analysis. Electrophysiological data were acquired with a Digidata 1330a interface and pClamp 10.4 software (both from Molecular Devices); data were analyzed using Clampfit 10.4 (Molecular Devices), OriginPro (OriginLab) and/or GraphPad Prism 7 (GraphPad). Numerical values presented here indicate mean ± SEM. Liquid-liquid junction potentials were estimated from the compositions of ionic species in the external and internal solutions, using the junction potential calculator in pClamp; membrane potentials stated in this paper include this correction.

### Electrophysiological Classification of Neuron Types

**Figure 1:**
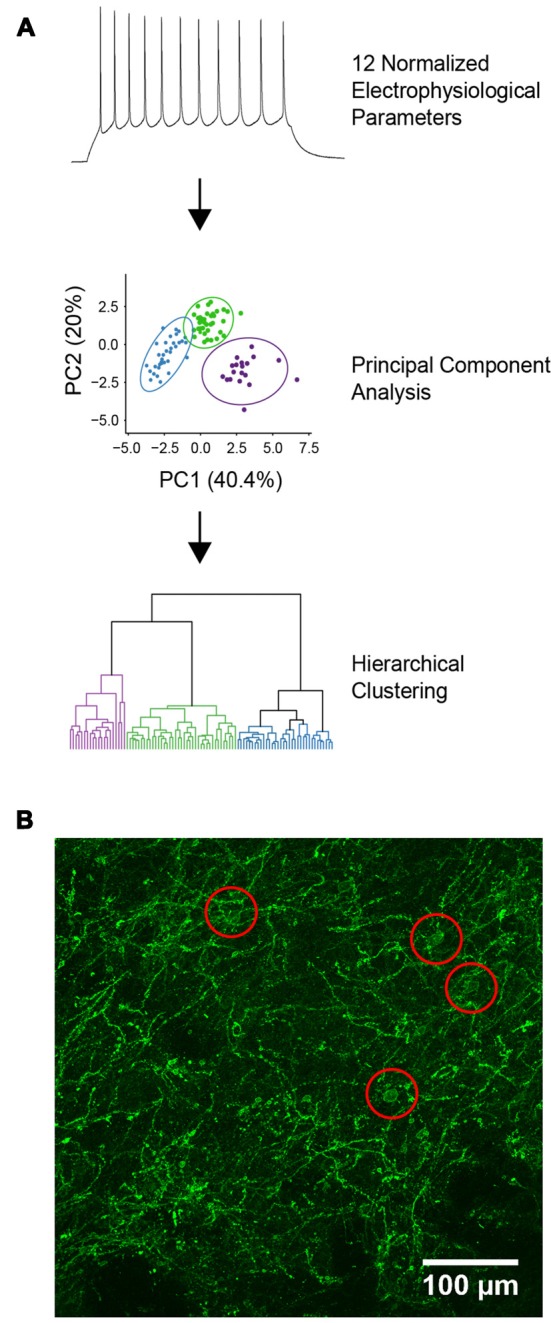
Characterization of YFP-positive neurons. **(A)** Diagram of the unbiased analytical methods used for cell classification. **(B)** Expression of channelrhodopsin-2 (ChR2)-YFP in the dorsal striatum of tyrosine hydroxylase (TH)-Cre × Ai32 mice. Red circles indicate locations of ChR2-YFP positive cell bodies.

To identify functionally related YFP^+^ neurons, intrinsic electrical properties were recorded from each cell and unsupervised hierarchical clustering was used to cluster these data (Figure 1A). A total of 12 electrical properties (Table 1) were determined from responses to a series of 1 s long current steps, ranging from −200 pA to 200 pA. Input resistance was calculated after constructing a current-voltage relationship from responses to hyperpolarizing current pulses. These responses were also used to determine other passive properties, such as membrane time constant, voltage sag during hyperpolarization, and input conductance. Responses to depolarizing current pulses were used to determine active properties, such as threshold current for evoking an AP, AP amplitude, after-hyperpolarization (AHP) amplitude, duration (half-width), and maximum rise and fall rates of APs. AP adaptation properties were measured from responses to the smallest current step that elicited at least 10 AP. These properties were extracted from raw data using custom scripts written in Matlab (Mathworks).

**Table 1:** Electrophysiological properties used for unbiased classification of YFP-positive neurons.

Property	Description
C_m_ (pF)	Membrane capacitance measured after whole-cell configuration was achieved.
R_in_ (MΩ)	Slope of linear fit between current-voltage response curve.
RMP (mV)	Y-intercept of linear fit between current-voltage response curve.
Sag amplitude (mV)	Difference between most hyperpolarized voltage and steady-state voltage, measured in response to hyperpolarizations to approximately −100 mV.
AHP (mV)	Difference in voltage from AP threshold to maximum negative deflection during repolarization.
AP amplitude (mV)	Difference between AP threshold and peak amplitude of 1st AP.
AP frequency adaptation	Ratio between instantaneous frequencies of the first two APs and last three APs.
AP amplitude adaptation	Ratio between amplitudes of first and last AP during AP train.
AP Half-width (ms)	Time difference between rising phase and decaying phase of AP at half-maximum amplitude.
Decay rate of AP (mV/ms)	Maximum rate of rise of membrane voltage during AP rising phase.
Rise rate of AP (mV/ms)	Maximum rate of rise of membrane voltage during AP falling phase.
Current threshold (pA)	Smallest current that evoked at least one AP.

From the 12 intrinsic properties, data dimensionality was reduced using principal component (PC) analysis. PCs were calculated using singular value decomposition, performed using the pcaMethods package in the R programming language (Stacklies et al., 2007; R Core Team, 2013). The first three PCs, which accounted for 69.2% of the total variance in the data, were used for unsupervised agglomerative hierarchical clustering using Euclidean distance as a measure of linkage distance. Ward’s (1963) minimum variance was used to combine clusters, which reduced the total within-cluster variance. This was implemented using the online webtool Clustvis, which uses R packages such as pheatmap, FactoMineR and ggplot2 internally to generate dendrograms of the hierarchically clustered data (Metsalu and Vilo, 2015). The optimal number of clusters was determined using silhouette analysis which measures the quality of clustering by calculating the separation between identified clusters (Rousseeuw, 1987). Silhouette analysis was performed using the cluster package in R with Euclidean distance used for calculating silhouette width (Maechler et al., 2018). To visualize the identified clusters, a dendrogram was constructed where the horizontal lines represent joined clusters. Radar plots were generated in R using the packages ggplot2 and radarchart after normalizing population feature means to ranges of 0–1.

### Immunohistochemistry and Imaging

Following electrical recordings, neurobiotin-filled cells were visualized by fixing brain slices in 4% paraformaldehyde overnight at 4°C followed by permeabilization in phosphate-buffered saline containing 0.25% Triton X-100 (PBST) for 1 h at room temperature. Slices were then incubated in streptavidin-Alexa 633 (1:1,000 diluted in PBST) for 48 h at 4°C before washing in PBST three times for 20 min each. Brain slices were mounted on glass slides and imaged using the same microscope system described above. A Z-stack of images was obtained for each labeled cell and cell morphology was digitally reconstructed as described below. Maximum projection images of such reconstructions are shown here unless otherwise stated.

To analyze and compare the morphology of neurobiotin-labeled neurons, cells were digitally reconstructed using AdReconstructor, a software suite for reconstruction of neuronal morphology (Nair et al., 2018). This program first applies pre-processing algorithms to remove common sources of noise, such as non-specific background staining and neurobiotin leakage. Cells were then reconstructed using the All Path Pruning algorithms developed by Xiao and Peng (2013) and post-processing measures were applied to ensure accuracy of reconstructions. The automated traces obtained in the open source SWC format were then quantified using L-Measure to obtain parameters such as surface area against path distance and spine density (Scorcioni et al., 2008). Sholl analysis was performed to analyze dendritic branching using the Simple Neurite Tracer plugin in ImageJ (Ferreira, 2016).

ChR2-YFP expressing cells in the dorsal striatum were also visualized by immunolabeling, using an anti-YFP/GFP antibody. The dendrites of YFP^+^ cells were dispersed throughout the dorsal striatum and the somata of these cells seemed to be evenly dispersed throughout the striatum (Figure 1B). Brain slices were fixed in 4% paraformaldehyde overnight at 4°C followed by permeabilization and blocking of the endogenous epitopes by applying 10% normal goat serum in PBST for 1 h at room temperature. Chicken anti-GFP antibody (Abcam) was applied at 1:1,000 in PBST for 48 h at 4°C and then washed three times in PBST for 20 min each. Goat anti-chicken Alexa 488 secondary antibody was applied at 1:500 in PBST for 2 h at room temperature followed by washing three times in PBST for 20 min each. All antibodies were obtained from Life Technologies unless otherwise stated. All image processing and analyses were carried out using FIJI, except those used for morphological reconstruction as described above.

### Morphological Classification of Neuron Types

To determine the relationship between electrophysiologically-defined cell types and their morphological properties, we also performed unsupervised hierarchical clustering of morphological features extracted from digital reconstructions of 15 neurobiotin-labeled neurons. The morphological properties extracted were based on definitions used in Scorcioni et al. (2008) and Gouwens et al. (2018), and are described in detail in Table 2. A total of 23 properties were extracted from digital reconstructions saved in the open source SWC format using the “compute global feature” plugin in Vaa3D (Peng et al., 2014). Dimensionality reduction was then performed using PC analysis, calculated using singular value decomposition and implemented using pcaMethods in R (Stacklies et al., 2007). Agglomerative hierarchical clustering was then performed on the first three PCs which account for 69.7% of the total variance in the dataset using the online tool Clustvis with correlation as a distance measure and Ward’s distances as a clustering method (Metsalu and Vilo, 2015). To identify the optimal number of clusters, silhouette analysis was again used- using Euclidean distance to calculate silhouette width- and implemented using the cluster package in R (Maechler et al., 2018). Radar plots were created in R using the packages ggplot2 and radarchart after normalizing population means to a range of 0–1.

**Table 2:** Morphological properties used for unbiased classification of YFP-positive neurons.

Property	Description
Number of nodes	The total number of nodes in the given digital reconstruction. A node in a reconstruction represents a single sample point of the neuron defined by its X, Y and Z coordinates, a radius, and its connectivity to other nodes in the neuron.
Soma surface area (μm^2^)	The surface of the spherical node representing the soma in the digital reconstruction.
Number of stems	The number of nodes attached to the soma.
Number of bifurcations	The number of points which have two daughter nodes for the given reconstruction.
Number of branches	The number of compartments that lie between two branching points or between one branching point and a termination point in the given reconstruction.
Number of tips	The number of terminal tips for the given input neuron.
Neuronal height (μm)	Height is the difference of minimum and maximum y-values after eliminating the outer points on either end by using the 95% approximation of the y-values of the given reconstruction.
Neuronal width (μm)	Width is the difference of minimum and maximum x-values after eliminating the outer points on either end by using the 95% approximation of the x-values of the given reconstruction.
Neuronal depth (μm)	Depth is the difference of minimum and maximum z-values after eliminating the outer points on either end by using the 95% approximation of the z-values of the given reconstruction.
Average diameter (thickness; μm)	The average diameter of all compartments of the neuron.
Total length (μm)	The total length of the neuron is computed as the sum of distances between two connected nodes for all branches.
Total volume (μm^3^)	The total volume of the entire neuron.
Maximum Euclidean distance to root (μm)	The maximum Euclidean distance of all nodes. Euclidean distance is the straight line distance from the soma to the node.
Maximum path distance to root (μm)	The maximum path distance of all nodes. The path distance is the sum of lengths of all connected nodes from the soma, ending with that node.
Maximum branch order	The maximum order of the branch. A branch’s order is defined with respect to the soma where the soma has a branch order = 0 and ever bifurcation has an increasing branch order.
Average contraction	The average ratio between Euclidean distance of a branch and its path length.
Average fragmentation	The average number of compartments that constitute a branch between two bifurcation points or between a bifurcation point and a terminal tip.
Average parent-daughter ratio	The average ratio between the diameter of a daughter branch and its parent branch
Average local amplitude angle (degrees)	The average angle between the first two compartments at a bifurcation
Average remote amplitude Angle (degrees)	The average angle between two bifurcation points or between bifurcation point and terminal point or between two terminal points.
Intersection at 30 μm	The number of Sholl plot intersections at a distance of 30 μm from the soma.
Intersection at 50 μm	The number of Sholl plot intersections at a distance of 50 μm from the soma.
Spine density (μm^−1^)	Total number of spines in all branches of a given neuron

## Results

### Three Types of YFP^+^ Striatal Neurons

**Figure 2:**
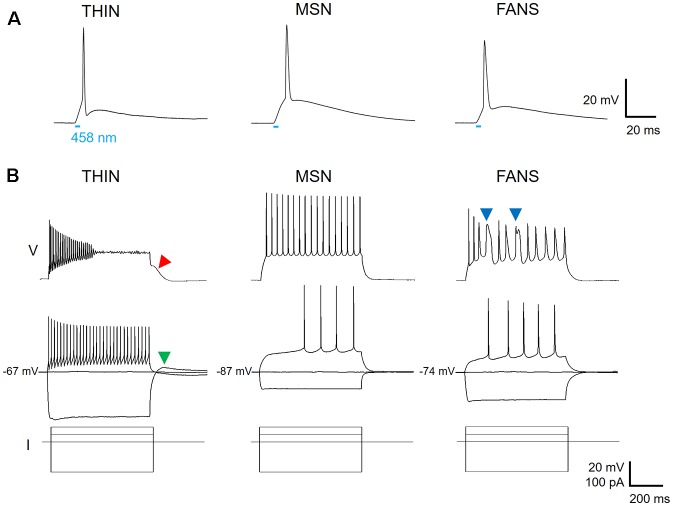
Three types of ChR2-YFP expressing cell in the dorsal striatum. **(A)** Action potentials could be evoked in response to a 5 ms laser light flash (458 nm, at bar), confirming expression of ChR2 in the three cell types. **(B)** Voltage responses to hyperpolarizing and depolarizing current pulses reveal different firing patterns for the three cell types (top two rows, current pulses shown at bottom).

In an attempt to identify TH-expressing neurons in the striatum, we used double-transgenic mice with ChR2 expression driven by the TH promoter. Positive striatal cells could be identified, in live brain slices prepared from these mice, by the fluorescence of YFP fused to the ChR2. Expression of ChR2 was confirmed by the APs that were evoked when brief (5 ms duration) laser light flashes (458 nm; 0.006–0.018 mW) were used to activate the ChR2 (Figure 2A). Due to the possibility of ectopic expression of ChR2-YFP in neurons that do not express TH in adulthood (Lammel et al., 2015; Stuber et al., 2015), we do not assume that neurons expressing ChR2-YFP actually express TH and instead will conservatively refer to these cells as YFP^+^ neurons.

**Figure 3:**
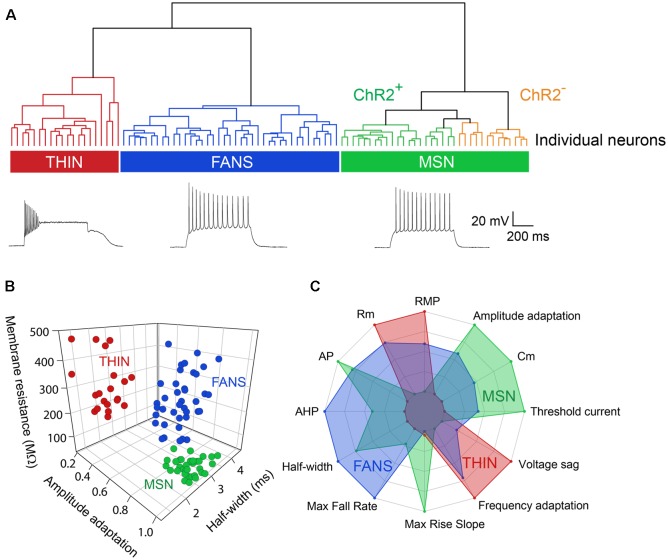
Unbiased hierarchical analysis revealed significant differences between the three types of YFP^+^ neurons. **(A)** A dendrogram showing the unbiased classification of the three types of YFP^+^ neurons (top) and spiking pattern of a representative cell from each type (bottom). **(B)** 3D scatter plots showing the clustering of the three YFP^+^ cell types. **(C)** Radar plots revealing substantial differences between the three cell types, with indicated properties displayed using normalized population means in the range of 0–1.

Applying depolarizing and hyperpolarizing current pulses to such YFP^+^ cells revealed a diversity of cellular responses (Figure 2B). Unsupervised hierarchical clustering of these cell responses, based on the 12 intrinsic electrical properties listed in Table 1, revealed that there were three different populations of YFP^+^ cells (Figure 3A). Comparing AP half-width, AP amplitude adaptation during prolonged depolarizations, and cell input resistance also showed a clear separation into the same three cell types identified by the clustering approach (Figure 3B). Indeed, all 12 intrinsic electrical properties considered differed significantly between the three different types of neurons (Figure 3C).

One type of cell resembled the THIN described previously (Ibáñez-Sandoval et al., 2010); these cells exhibited high input resistance and prominent voltage sag during responses to hyperpolarizing currents (Figure 2B, left and Figure 3C). THIN often exhibited a rebound depolarization following a hyperpolarizing current pulse (Figure 2B, green arrowhead), which sometimes evoked rebound APs. Large depolarizing current pulses often evoked damped voltage oscillations during such depolarizations, as well as a plateau potential following the end of depolarization (Figure 2B, red arrow). Four subtypes of THIN, with subtle differences in their electrophysiological properties, have been reported (Ibáñez-Sandoval et al., 2010), and these are evident in the subclusters of THIN shown in Figure 3A. To simplify our analyses, we have grouped together recordings made from all of these THIN subtypes.

**Figure 4:**
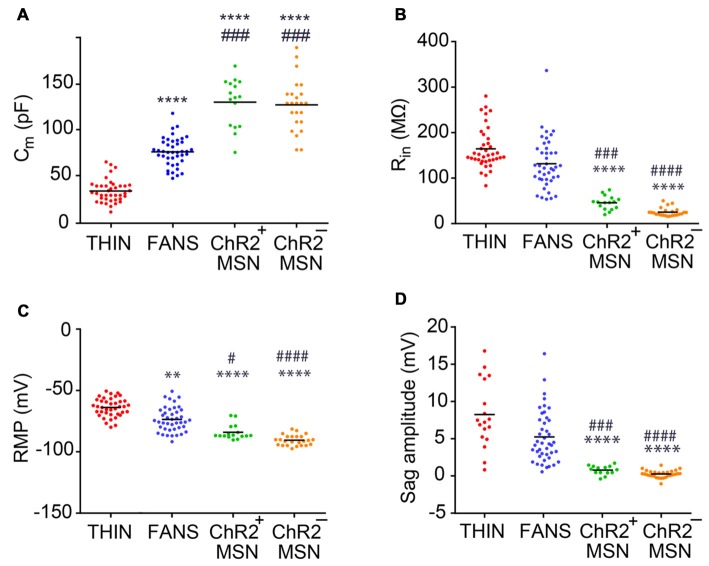
Passive electrical properties of the YFP^+^ neurons in the dorsal striatum. **(A)** Membrane capacitance. **(B)** Input resistance. **(C)** Resting membrane potential (RMP). **(D)** Amplitude of voltage sag during responses to hyperpolarizing current. One-way ANOVA with Dunn’s multiple comparison test; ^#^*p* < 0.05, **p < 0.01, ^###^*p* < 0.001, ****^/####^*p* < 0.0001, where *and ^#^indicate comparisons with THIN and FANS, respectively. Each horizontal line indicates mean value.

Unexpectedly, a small proportion of the YFP^+^ cells unambiguously exhibited the intrinsic electrical properties of MSN, specifically low input resistance and delayed spiking with depolarization (Figure 2B, middle and Figure 3C). While these cells exhibited some small differences in population means when compared to ChR2^−^ MSN, such as their higher RMP and input resistance, as well as smaller AP and AHP amplitudes (see Figure 4), overall in our analysis they clustered together with MSN that did not express ChR2 (Figure 3A). This indicates that the two populations of MSN are much more similar to each other than they are to the other YFP^+^ cell types.

In addition, a third population of cells displayed properties distinct from those of any striatal cell described previously. These cells were characterized by intrinsic electrical properties that were intermediate between those of THIN and MSN (Figures 3B,C). Although their responses to small depolarizing currents were more similar to those of MSN than THIN (Figure 2B, right), they exhibited significantly more AP adaptation during prolonged depolarizations (see Figures 6–8 below). Further, in response to large depolarizations, these cells exhibited APs that broadened substantially, often yielding plateau potentials (Figure 2B, blue arrows). As will be established in more detail below, these cells are unique in their properties, most notably their large range of AP frequency adaptation (see Figure 6) and the presence of spines on their dendrites (see Figure 9). We have therefore named these cells Frequency-Adapting Neurons with Spines (FANS).

**Figure 5:**
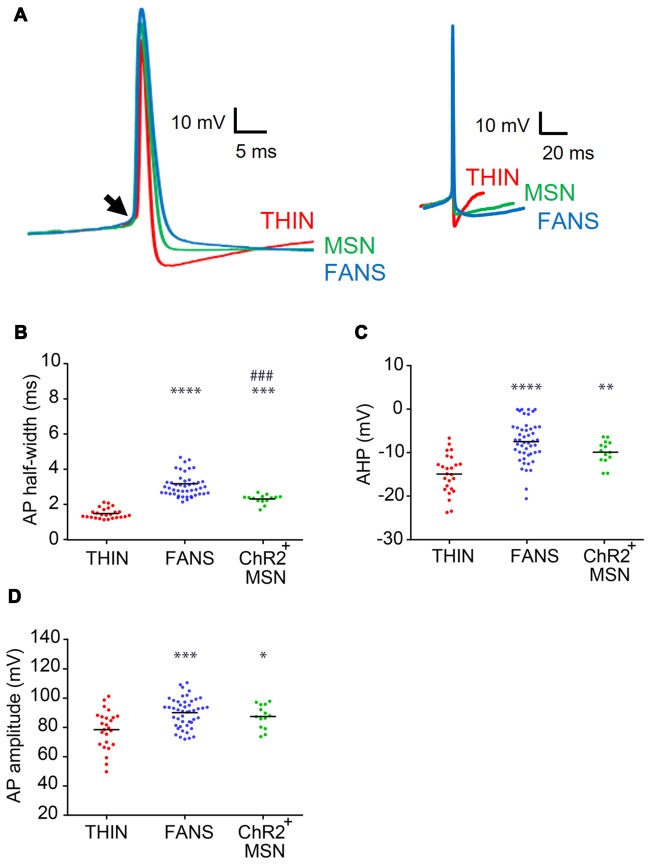
AP characteristics of YFP^+^ neurons in the dorsal striatum. **(A)** Representative APs recorded from each cell type, shown at fast (left) and slow (right) time scales. Arrow indicates the point of inflection of APs, which was used as the reference point when measuring the amplitudes of APs and their after-hyperpolarizations (AHPs). **(B)** AP duration as measured at half-width. **(C)** AHP amplitude. **(D)** AP peak amplitude. One-way ANOVA with Holm-Sidak’s multiple comparisons; *p < 0.05, **p < 0.01, ***^/####^*p* < 0.001, ****p < 0.0001, where *and ^#^indicate comparisons with THIN and FANS, respectively. Each horizontal line indicates mean value.

**Figure 6:**
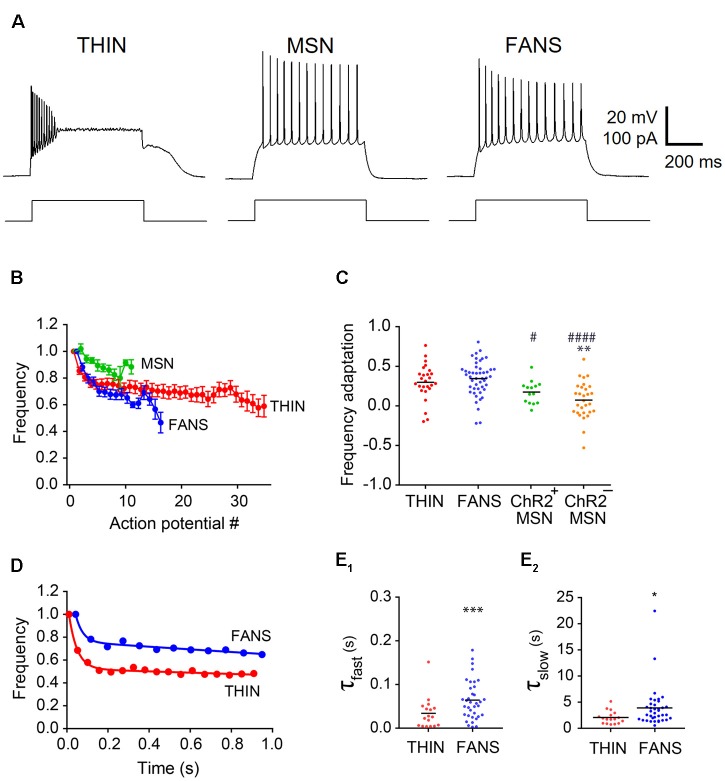
Adaptation of AP frequency in YFP^+^ neurons. **(A)** Representative traces of APs evoked by 1 s current pulses. **(B)** Normalized mean instantaneous frequency (IF); error bars indicate SEM. **(C)** Frequency adaptation calculated as (1-last AP/first AP). One-way ANOVA with Dunn’s multiple comparisons; *^/#^*p* < 0.05, **p < 0.01 and ^####^*p* < 0.0001, where *and ^#^indicate comparisons with THIN and FANS, respectively. **(D)** Normalized IF vs. time plots fitted by two-component exponential curve from representative THIN and FANS neurons. **(E)** Exponential fit time constants (τ) of the fast **(E1)** and slow **(E2)** components of THIN and FANS calculated for IF (unpaired *t*-tests; ***p < 0.001; ****p < 0.001). Each horizontal line indicates mean value.

**Figure 7:**
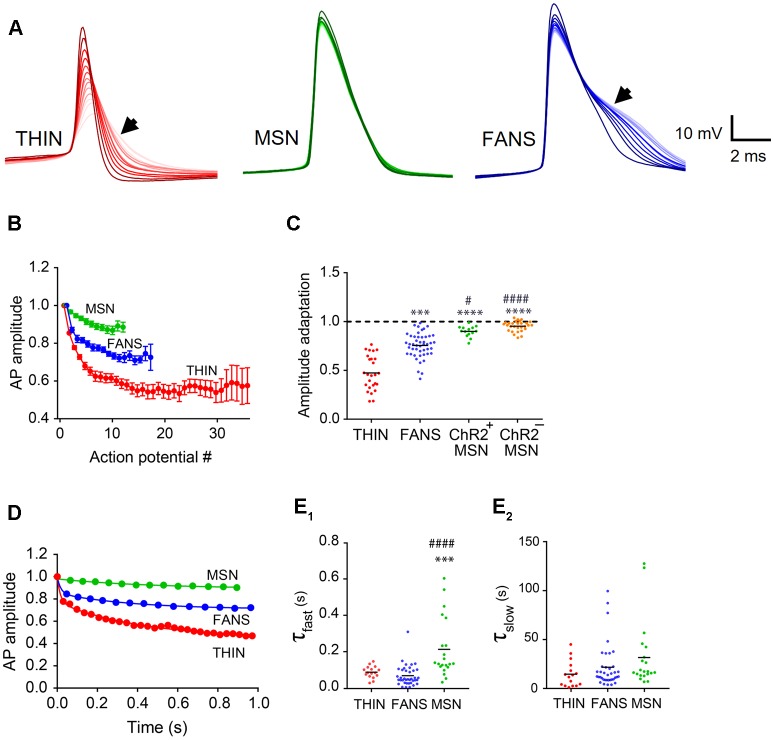
AP amplitude adaptation in YFP^+^ neurons of the dorsal striatum. **(A)** Representative traces of APs. Dark to light colors indicate AP progression. Arrows indicate AP broadening evident in THIN and FANS. **(B)** Normalized mean AP peak amplitude, mean ± SEM. **(C)** AP amplitude change calculated as the ratio of last AP/first AP. One-way ANOVA with Dunn’s multiple comparisons; ^#^*p* < 0.05, ***p < 0.001 and ****^/####^*p* < 0.0001, where *and ^#^indicate comparisons with THIN and FANS, respectively. **(D)** Normalized amplitude vs. time plots fitted by two-phase exponential curve from representative cells. **(E)** Exponential fit time constants (τ) of the fast **(E1)** and slow **(E2)** components of THIN, FANS and ChR2^+^MSN calculated for AP amplitude (one-way ANOVA with Tukey’s multiple comparisons; ***p < 0.001, ^####^*p* < 0.0001). Each horizontal line indicates mean value.

**Figure 8:**
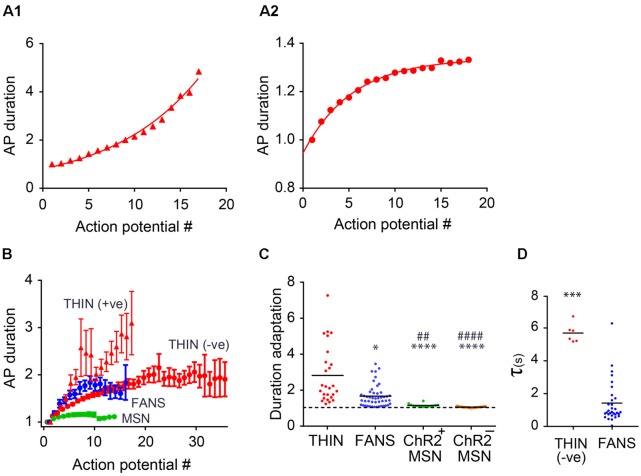
AP duration adaptation in YFP^+^ neurons in the dorsal striatum. **(A)** Changes in AP duration (measured at half-width) from a representative THIN showing positive **(A1)** and negative **(A2)** curvatures. **(B)** Normalized mean AP duration, mean ± SEM. **(C)** AP duration adaptation calculated as the ratio of last AP/first AP. One-way ANOVA with Dunn’s multiple comparisons; *p < 0.05, ^##^*p* < 0.01 and ****^/####^*p* < 0.0001, where *and ^#^indicate comparisons with THIN and FANS, respectively. **(D)** Time constants (τ) of AP broadening for THIN and FANS (unpaired *t*-test; ***p = 0.0009). Each horizontal line indicates mean value.

**Figure 9:**
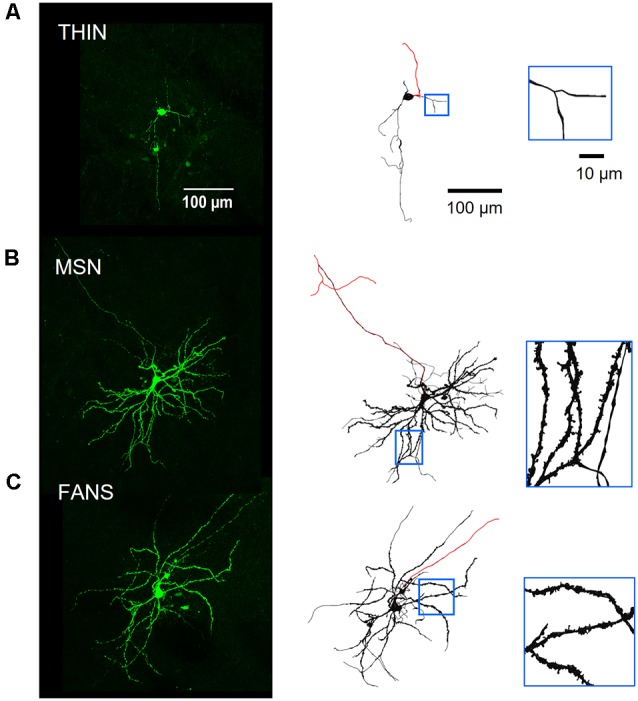
Morphology of neurobiotin-filled YFP^+^ striatal neurons. Panel **(A)** shows a THIN, while **(B)** shows a MSN and **(C)** is a FANS. Left—Green-on-black images are maximum-projection fluorescence images. Right—Black-on-white images are 3D-reconstructed neurons. Red lines indicate axons, distinguished by their smaller diameters and absence of spines. Regions in blue boxes are enlarged in the insets to reveal the presence of dendritic spines in both MSN and FANS, but not in THIN.

### Intrinsic Electrical Properties of Three YFP^+^ Cell Types

We characterized the three types of YFP^+^ neurons more extensively by comparing their passive electrical properties (Figure 4). We first measured the apparent membrane capacitance (C_m_), which is proportional to cell surface area and, hence, the overall size of the cell. The C_m_ of the three YFP^+^ cell types were significantly different from each other (Figure 4A; one-way ANOVA, *p* < 0.0001; Dunn’s multiple comparison test), with THIN being smallest, followed by FANS and MSN. The C_m_ of YFP^+^ MSN was very similar to that of YFP^−^ MSN. As expected, cell input resistance—which is inversely related to surface area—followed the reverse order, with THIN and FANS significantly different from MSN but not from each other (Figure 4B; *p* < 0.0001; one-way ANOVA with Dunn’s multiple comparison test). Again, there were no significant differences between the input resistances of MSN that did or did not express ChR2-YFP.

Striatal MSN are characterized by hyperpolarized RMPs and this was evident both in MSN that did or did not express ChR2-YFP (Figure 4C). In contrast, THIN and FANS had RMPs that were significantly more depolarized than those of MSN (*p* < 0.0001; one-way ANOVA with Dunn’s multiple comparison test). In addition, the amplitude of the depolarizing sag in membrane potential in response to hyperpolarizing current pulses—when measured at similar membrane potentials to take into account differences in input resistance (see Figure 2A, left)—was largest in THIN (Ibáñez-Sandoval et al., 2010) while FANS had an intermediate degree of sag and MSN exhibited the smallest amount of sag (Figure 4D; *p* < 0.0001; one-way ANOVA with Tukey’s multiple comparison test). In summary, the passive electrical characteristics (and amount of depolarizing sag) of THIN, FANS and MSN were distinctively different from each other.

We next compared the waveforms of APs evoked in the three types of YFP^+^ cells in response to depolarizing current pulses (Figure 5A). FANS had the broadest APs (Figure 5A, left), measured as their width at half-maximum amplitude, while THIN had the narrowest APs (Figure 5B; *p* < 0.0001; one-way ANOVA with Holm-Sidak’s multiple comparison test). The reverse was true for the AHP that followed the APs (Figure 5A, right): AHP magnitudes were greatest for THIN while FANS and MSN were similar (Figure 5C; *p* < 0.0001; one-way ANOVA with Holm-Sidak’s multiple comparison test). Peak AP amplitude, measured for the first AP evoked during a depolarizing current pulse, was largest for FANS and smallest for THIN (Figure 5A, left); the APs of MSN were similar in amplitude to those of FANS (Figure 5D; *p* = 0.0002; one-way ANOVA with Holm-Sidak’s multiple comparison test).

### Action Potential Adaptation During Sustained Depolarization

During trains of APs, AP properties dynamically changed due to adaptation (Figure 6A). We quantitatively compared AP adaptation in response to sustained depolarizations (1 s duration) of all three types of YFP^+^ striatal cells. Because adaptation properties vary with the degree of depolarization, we measured adaptation in different cells at a standard criterion current level of 50 pA above the minimum current required to evoke a single AP.

#### Frequency Adaptation

The frequency of APs declined during sustained depolarizations (Figure 6A). Plots of normalized AP instantaneous frequency (IF), defined as the inverse of the interval between successive APs, illustrate the differences in the AP frequency adaptation properties of these three cell types (Figure 6B). While both FANS and THIN showed substantial AP frequency adaptation, evident as a progressive decline in IF during a train of APs, MSN exhibited less frequency adaptation. The degree of decline during these trains was quantified by the ratio of IF of APs measured at the beginning and end of the depolarizing current pulse (Figure 6C). MSN showed significantly less AP frequency adaptation in comparison to THIN or FANS (*p* < 0.0001; one-way ANOVA with Dunn’s multiple comparison test).

In most THIN and FANS, the time course of the decline in AP frequency during adaptation could be described by a double exponential function, with a fast initial phase followed by a second, more gradual component (Figure 6D). When a plateau potential occurred in FANS, AP frequency adaptation was interrupted and appeared to reset. For this reason, responses that included plateau potentials were excluded from our analysis. MSN were also excluded, because they exhibited relatively little AP frequency adaptation. The mean time constants of both the fast and slow components for the remaining FANS were significantly larger than those of THIN, indicating slower frequency adaptation in FANS (Figures 6E1,E2; *p* < 0.001, unpaired *t*-test).

#### Amplitude Adaptation

In all three cell types, the peak amplitudes of APs also progressively decreased during prolonged depolarization (Figures 6A, 7A). The kinetics of this adaptation of AP amplitude is illustrated for all three cell types in Figure 7B. By calculating the ratio of the amplitudes of the last and first APs, we could compare the amount of this adaptation of AP amplitude across the three cell types (Figure 7C). THIN exhibited the greatest amount of AP amplitude adaptation, while MSN had the least (Figure 7C; *p* < 0.0001; one-way ANOVA with Dunn’s multiple comparison test). For all three cell types, AP amplitude adaptation followed a bi-exponential time course (Figure 7D). Calculation of the time constants for these exponential functions indicated that the initial rate of adaptation was similar in THIN and FANS, while MSN had significantly slower adaptation (Figure 7E1; *p* < 0.0001, one-way ANOVA with Tukey’s multiple comparison test). The three cell types showed no significant differences in the kinetics of the slow phase of amplitude adaptation, although there was a tendency for THIN to have slightly faster adaptation than FANS and for FANS to be slightly faster than MSN (Figure 7E2; *p* = 0.1158, one-way ANOVA).

#### AP Broadening

During prolonged depolarizations, AP duration also progressively broadened in both FANS and THIN (Figure 7A, black arrows). Two temporal patterns for AP broadening were observed for different THIN: accelerating (positive curvature; Figure 8A1) or decelerating (negative curvature; Figure 8A2) changes in the rate of adaptation, indicated by the slopes of the plots. FANS also tended to exhibit decelerating broadening of APs (Figure 8B). The degree of AP broadening measured at half-width was on average larger for THIN than for FANS (Figure 8C). In contrast, MSN exhibited little, if any, AP broadening (*p* < 0.0001; one-way ANOVA with Dunn’s multiple comparison test). Due to the difference in the kinetics of AP broadening in THIN, only THIN with negative curvature were compared with FANS. In these cells, the time course of AP broadening could be described by single exponential functions. The rate of adaptation of AP duration in THIN was slower than in FANS, as demonstrated by the larger time constants for AP broadening in THIN (Figure 8D; unpaired *t*-test, *p* = 0.0009).

In summary, FANS exhibit distinctive electrophysiological properties that are uniquely different from the properties of both THIN and MSN (Table 3). Therefore, FANS appear to be a novel, previously unrecognized cell type within the striatum.

**Table 3:** Electrical and morphological properties of TH interneuron (THIN), frequency-adapting neurons with spines (FANS) and medium-sized spiny neuron (MSN).

Property	THIN	FANS	MSN (ChR2^+^)	MSN (ChR2^−^)
C_m_ (pF)	34.7 ± 2.0	76.6 ± 2.4	131 ± 6.5	128 ± 5.7
R_in_ (MΩ)	328 ± 14.5	262 ± 17.0	90.8 ± 7.6	49.6 ± 3.8
RMP (mV)	−63.9 ± 1.2	−73.6 ± 1.5	−84.1 ± 1.5	−90.6 ± 0.9
Sag amplitude (mV)	4.4 ± 0.8	1.5 ± 0.2	0.5 ± 0.1	0.4 ± 0.1
AP duration (ms)	1.47 ± 0.06	3.20 ± 0.14	2.34 ± 0.07	
AHP (mV)	−14.9 ± 0.9	−7.4 ± 0.7	−10.0 ± 0.7	
AP amplitude (mV)	78.7 ± 2.6	90.2 ± 1.5	87.6 ± 2.1	
AP frequency adaptation	0.30 ± 0.04	0.34 ± 0.03	0.18 ± 0.04	0.08 ± 0.04
AP duration adaptation	2.82 ± 0.31	1.68 ± 0.09	1.14 ± 0.02	1.01 ± 0.01
AP amplitude adaptation	0.47 ± 0.03	0.76 ± 0.02	0.90 ± 0.01	0.95 ± 0.01
Soma diameter (μm)	13.7 ± 0.5	15.5 ± 0.8	13.8 ± 1.0
No. primary dendrites	3.3 ± 0.3	3.9 ± 0.3	5.5 ± 0.2	
Spine density (μm^−1^)	0	0.52 ± 0.04	0.57 ± 0.06	

*Values indicate mean ± SEM*.

### Unique Morphology of FANS

To examine their morphology, YFP^+^ neurons also were filled with neurobiotin during whole-cell patch clamp recordings (Figure 9, left). Neurobiotin-filled neurons were then fixed, processed and imaged on a two-photon microscope. Stacks of cell images obtained at different z-axis planes were then digitally reconstructed in an automated fashion to obtain bias-free representations of neuronal morphology (Figure 9, right; see “Materials and Methods” section). THIN had somata that were medium-sized (13.7 ± 0.5 μm, long axis diameter) and oval or round in shape (Figure 9A). The primary and secondary dendrites of THIN appeared to branch sparsely and were often varicose; most notably, their dendrites lacked spines (Figure 9A, inset). These morphological features are very similar to those previously reported for THIN (Ibáñez-Sandoval et al., 2010). The morphology of YFP^+^ MSN also resembled typical MSN (Gertler et al., 2008; Kreitzer, 2009): they had medium-sized somata (13.8 ± 1.0 μm, long axis diameter) and dendrites that branched extensively and were densely covered with spines (Figure 9B). Occasionally the main axon of these cells could be observed for a sufficient length to visualize its projection towards the posterior of the brain. The morphology of FANS was different from that of THIN or MSN: the dendrites of FANS were also covered with spines, similar to MSN, but their dendritic branching was much less extensive (Figure 9C). The somata of FANS were also medium-sized (15.5 ± 0.8 μm, long axis diameter) and were mostly oval in shape.

**Figure 10:**
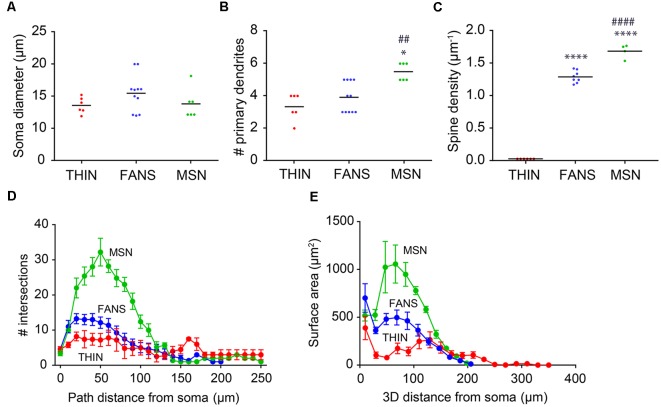
Morphological analysis of YFP^+^ striatal neurons. **(A)** Soma diameter (one-way ANOVA, *p* = 0.2458; Dunn’s multiple comparisons). **(B)** Number of primary dendrites (one-way ANOVA, *p* = 0.0031; Dunn’s multiple comparisons; *p < 0.05 to THIN, ^##^*p* < 0.01 to FANS). **(C)** Spine density (one-way ANOVA, *p* < 0.0001; Tukey’s multiple comparisons; ****p < 0.0001 to THIN, ^####^*p* < 0.0001 to FANS). **(D)** Sholl analysis indicating dendritic arborization, with points indicating means and error bars indicating ±SEM. **(E)** Surface area vs. path distance plot to indicate dendritic branching, with points indicating means and error bars indicating ±SEM.

Several of the morphological properties of the three types of YFP^+^ neurons are quantified in Figure 10. All three cell types had similar-sized soma (Figure 10A; *p* = 0.25, one-way ANOVA with Dunn’s multiple comparison test). MSN had significantly more primary dendrites than either FANS or THIN (Figure 10B; *p* = 0.0031, one-way ANOVA with Dunn’s multiple comparison test). MSN also had the highest density of dendritic spines, as revealed by a 3D analysis of spine density from the reconstructed neurons (Figure 10C). FANS had a lower density of spines compared to MSN, while THIN had no spines (*p* ≤ 0.001, one-way ANOVA with Tukey’s multiple comparison test). Our measured values of spine density for MSN are similar to those previously reported (Shen et al., 2008).

Dendritic branching in the three types of cells first was compared by a two-dimensional analysis described by Sholl (1953). This analysis revealed that dendritic branching was greatest in MSN and least in THIN (Figure 10D). Although the extent of branching was similar in MSN and FANS within the first 10 μm from their somata, MSN branched more extensively at 20–130 μm from their somata (Figure 10D; *p* < 0.05; multiple *t*-tests). As an alternative way to compare dendritic branching in the three cell types, a three-dimensional variant of the Sholl analysis was also performed (Scorcioni et al., 2008). This analysis measured neurite surface area as a function of path distance from the cell body and captured the spatial extent of the neuron in three-dimensions, as opposed to the planar projections used in the Sholl (1953) analysis. Furthermore, measurement of surface area increases the functional relevance of the analysis because surface area should be more proportional to the number of synapses. This analysis (Figure 10E) revealed the same differences between the MSN and FANS that were evident in the Sholl (1953) analysis (Figure 10D), with MSN having the most dendritic branching, THIN having the least and FANS exhibiting branching intermediate between MSN and THIN over the first 100 μm from the cell body (*p* < 0.05, multiple *t*-tests).

**Figure 11:**
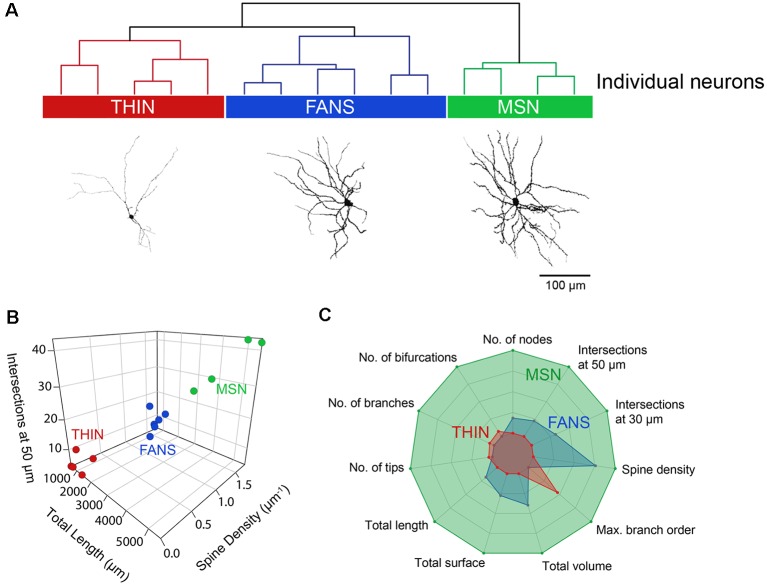
Unsupervised hierarchical clustering of morphology recapitulates electrophysiologically defined cell types. **(A)** A dendrogram showing the unbiased classification of the three types of YFP^+^ neurons (top) and digital reconstruction of a representative cell from each type (bottom). **(B)** 3D scatter plots showing the clustering of the three YFP^+^ cell types. **(C)** Radar plots revealing substantial differences between the three cell types, calculated using normalized population means in the range of 0–1.

To systematically examine the relationship between the morphological properties of the three types of YFP^+^ neurons, we extracted 23 features from digital reconstructions of these neurons and then performed an unsupervised clustering analysis. FANS clustered separately from both THINs and MSNs in our analysis (Figure 11A); remarkably, although the intrinsic electrical properties of these neurons were not considered in the morphological clustering analysis of Figure 11A, the clustering nonetheless completely segregated the neurons in a way that was consistent with the distinctive electrical properties of these neurons. This indicates that FANS represent a unique cell type both in regard to their morphological properties and their intrinsic electrical properties. Comparing dendritic branching within the first 50 μm from the soma, total length and spine density also showed a clear separation into the same three cell types identified by electrophysiology (Figure 11B). In fact, 11 of the morphological properties that we considered differed considerably between the three different types of YFP^+^ neurons, with a fairly consistent trend that MSNs possessed the highest means for each of these features, followed by FANS and then THIN (Figure 11C).

### Heterogeneity of FANS

**Figure 12:**
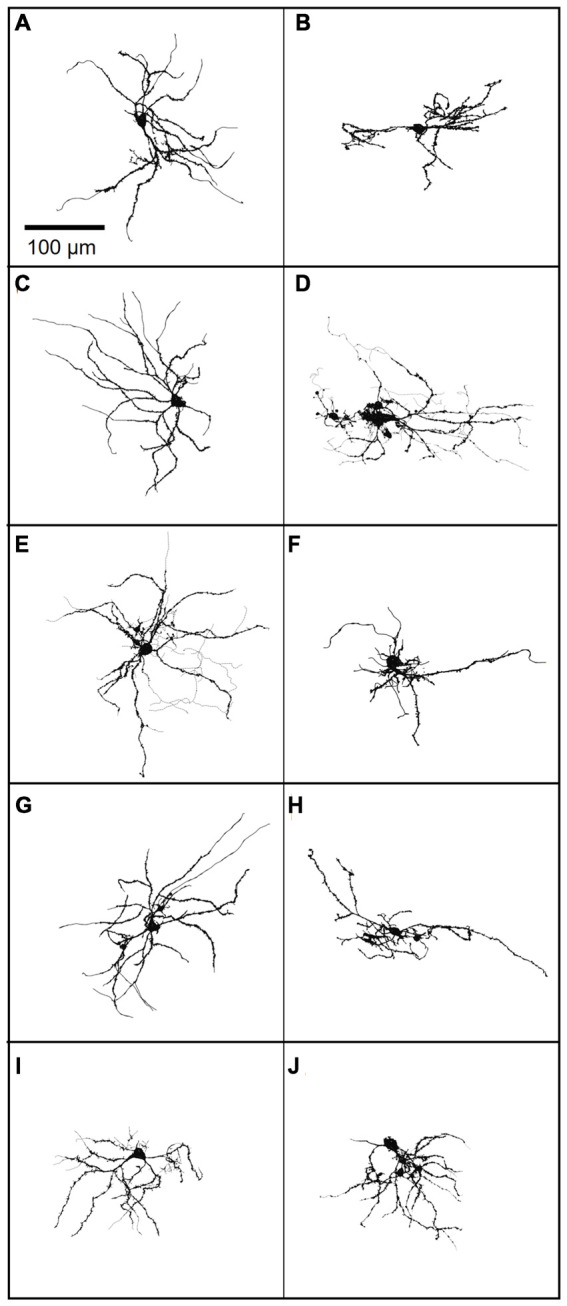
**(A–J)** A gallery of 10 neurobiotin-filled FANS showing their diverse morphology. Light lines indicate axons, wherever traceable; dark lines indicate dendrites.

Although FANS were distinctly different from THIN and MSN in regard to their intrinsic electrical properties and morphology, there was considerable heterogeneity between individual FANS. FANS were diverse both in terms of their dendritic field orientation and in their AP firing patterns. For example, their dendritic fields could be oriented along the long axis of the cell body, towards one side of the cell body, or could radiate in numerous directions around the soma (Figure 12).

**Figure 13:**
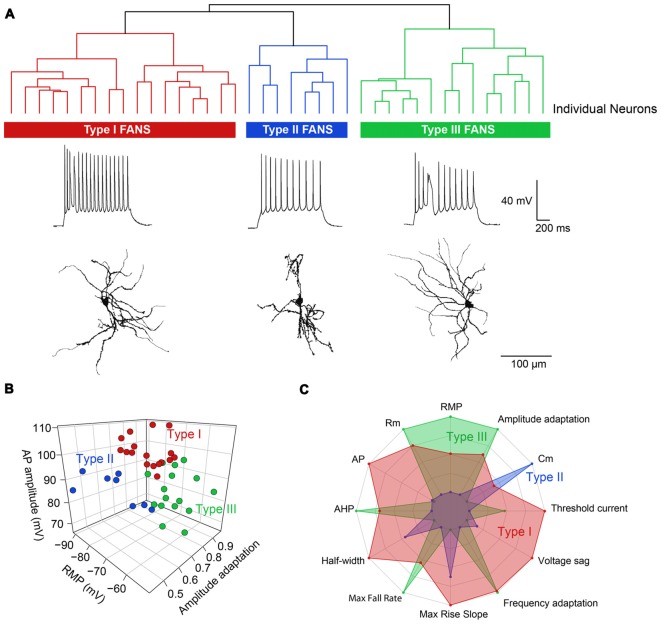
Heterogeneous morphological and functional properties of FANS. **(A)** Dendrogram revealing three subtypes of FANS. A representative cell’s morphology and spiking pattern are shown for each subtype. **(B)** 3D scatter plot showing the three most distinct electrophysiological properties between the three subtypes. **(C)** Radar plot summarizing the overall differences between the three subtypes, calculated using normalized population means in the range 0–1.

Hierarchical clustering, based on intrinsic electrical properties, revealed three subtypes of FANS (Figure 13A). We refer to these as Types I, II and III. The three subtypes of FANS showed differences in their AP amplitude, RMP and amount of AP amplitude adaptation (Figure 13B). These FANS subtypes also showed differences in most of the 12 properties used for classification (Figure 13C and Table 4). The temporal patterns of AP firing varied between the different classes of FANS, with depolarization-induced plateau potential present mostly in Type III FANS. Due to the relatively small number of FANS successfully filled with neurobiotin, it is not yet clear whether the differences in morphology and intrinsic electrical properties of FANS are correlated. However, based on an analysis of the 10 neurobiotin-filled FANS shown in Figure 12, it appeared that Type II FANS had less dendritic arborization than the other two FANS subtypes (Figure 13A, bottom).

**Table 4:** Electrical properties of Type I, II and III FANS.

Property	Type I	Type II	Type III
C_m_ (pF)	76.8 ± 3.4	81.7 ± 7.0	73.3 ± 3.1
R_in_ (MΩ)	275 ± 21	164 ± 18	312 ± 29
RMP (mV)	−74.0 ± 1.7	−82.1 ± 2.3	−66.2 ± 2.1
Sag amplitude (mV)	1.8 ± 0.5	1.4 ± 0.3	1.3 ± 0.1
AP duration (ms)	3.3 ± 0.1	3.0 ± 0.2	2.8 ± 0.1
AHP (mV)	−3.7 ± 0.6	−5.1 ± 1.7	−3.1 ± 0.9
AP amplitude (mV)	100 ± 1.3	83.6 ± 2.9	83.1 ± 2.1
Maximum AP frequency adaptation (Hz)	45.8 ± 6.3	26.6 ± 14	46.3 ± 6.7
AP amplitude adaptation	0.75 ± 0.02	0.63 ± 0.04	0.82 ± 0.02
Current threshold (pA)	43.5 ± 2.5	27.5 ± 6.4	35.0 ± 4.6

*Values indicate mean ± SEM*.

## Discussion

Here, we describe a heterogeneous population of YFP^+^ neurons in the striatum of transgenic mice with ChR2-YFP expression driven by the TH promoter.

### Heterogeneity of YFP^+^ Striatal Neurons

Three main subtypes of YFP^+^ striatal neurons were found with distinct morphological and electrophysiological properties (Figures 2, 3; Table 3). One subtype resembled the THIN described previously (Ibáñez-Sandoval et al., 2010), confirming the existence of these cells *via* a different genetic targeting strategy. Most THIN we examined were of the Type I or III described previously (Ibáñez-Sandoval et al., 2010). In fact, these two THIN subtypes are very similar to each other in terms of their firing patterns; they mainly differ in their RMP and input resistances. A second, small proportion of neurons resembled MSN. While this could arise from ectopic expression of Cre recombinase, as has been previously reported in some TH-Cre lines (Lammel et al., 2015; Stuber et al., 2015), it is also possible that these MSN might express TH at some point during their development. A third distinct population of YFP^+^ striatal neurons has not been observed previously in mature animals; these neurons have morphological and electrophysiological properties that uniquely distinguish them from all other YFP-expressing neurons (Figure 3). We have named these cells FANS. FANS have passive membrane properties (C_m_, R_in_, RMP and sag amplitude) that are intermediate between THIN and MSN (Figure 4) and their AHPs are longest lasting and smallest in amplitude (Figure 5). Similar to THIN, FANS show substantial adaptation of AP frequency, duration and peak amplitudes during AP trains, but differ from THIN in the amount or rate of adaptation (Figures 6–8). Morphologically, FANS have dendritic spines; while MSN are the only striatal neurons previously described to have dendritic spines, our quantitative analysis indicates that FANS spine density and dendritic branching are significantly lower than that of MSN (Figures 9–11). Finally, FANS may represent a heterogeneous population because they have diverse morphological and electrophysiological properties (Figures 12, 13).

### Unique Properties of FANS

Our discovery of FANS is surprising because they were not reported in previous descriptions of THIN in TH-EGFP mice (Grace and Bunney, 1983; Ibáñez-Sandoval et al., 2010; Ünal et al., 2011, 2015) or in TH-Cre mice with virally expressed ChR2-EYFP (Xenias et al., 2015). The reason why FANS were not observed previously is unknown but could be related to their relatively low numbers and the absence of specific markers to identify them. In addition, transgene expression patterns may differ with the same promoter due to positional effect variegation. Finally, it could be that TH-expressing cells could have been underreported in previous experiments; for example, in the experiments of Xenias et al. (2015), not all dopamine-expressing neurons in the midbrain expressed EYFP. The electrophysiological properties of FANS are somewhat similar, but not identical, to those of the type A TH-expressing striatal neurons described in neonatal mouse striatum by Masuda et al. (2011). Likewise, THIN are similar to what these authors call type B neurons. Thus, it is possible that these two types of TH-expressing neurons in neonatal striatum are the developmental precursors of FANS and THIN in the adult.

FANS are not a subtype of THIN, because their firing patterns do not resemble any previously described THIN subtypes. In addition, FANS have dendrites with a high density of spines, while THIN are largely aspiny; only a very small proportion of Type I THIN reportedly have spine-like appendages and the density of these structures still is much lower than the density of dendritic spines we observed in FANS (Ibáñez-Sandoval et al., 2010).

Two lines of evidence clearly indicate that FANS also are not MSN. First, none of the 12 electrophysiological and 11 morphological properties of FANS clustered with MSN in our unbiased clustering analyses (see Figures 3 and 11). Second, we did not encounter any FANS amongst non-YFP^+^ neurons of the striatum; most non-YFP^+^ neurons we examined were MSN while we occasionally encountered PV and cholinergic interneurons. With MSN making up ~95% of the total striatal population, we would expect to see FANS relatively frequently among non-YFP^+^ neurons if they are indeed a subtype of MSN. Finally, in contrast to the axons of MSN, we never observed the axons of FANS projecting out of the striatum. Therefore, we are confident that FANS are distinctly different from MSN.

FANS are also unlikely to be the other types of striatal interneurons that have been previously identified (see Tepper et al., 2010 for a review of striatal interneurons). For example, FANS do not exhibit the rapid APs that characterize PV interneurons. They also lack the low-threshold calcium spikes observed in SOM/NPY/NOS-positive interneurons and have much lower input resistance than these interneurons. Further, cholinergic interneurons have very large cell bodies, tend to fire spontaneous APs and have prominent hyperpolarization-induced sag, all properties that differ from those of FANS. Most importantly, unlike striatal interneurons, FANS have spines. We therefore conclude that FANS are a unique type of striatal neuron.

The plateau spikes generated in FANS in response to large depolarizing currents resemble the complex spikes seen in cerebellar Purkinje cells and cartwheel cells of the dorsal cochlear nucleus, two electrical signals that arise from activation of voltage-dependent calcium channels (Llinás and Sugimori, 1980; Kim and Trussell, 2007; Portfors and Roberts, 2007). Based on these similarities, we speculate that the depolarization-induced plateau spikes, as well as the other distinctive intrinsic properties, may allow FANS to provide input with a unique temporal structure in comparison to other striatal neurons. In addition, because FANS have dendritic spines, it is likely that they receive glutamatergic input from the cortex and/or dopaminergic input from the midbrain; both of these types of input contact dendritic spines on MSN (Freund et al., 1984; Dubé et al., 1988).

Several questions remain regarding the identity of FANS in the striatum. First, although we used TH-Cre mice as a tool for genetic access to FANS, it is not clear whether or not FANS express TH. It is possible that FANS do express TH at some time during their development or FANS may not express TH at all; as mentioned above, ectopic expression has been reported in TH-Cre mouse lines (Lammel et al., 2015; Stuber et al., 2015). Future work will be needed to understand why Cre (and ChR2-YFP) is expressed in FANS in our mice. Second, it is not known what neurotransmitter(s) FANS use. For the case of GABAergic THIN, despite the expression of TH these neurons are not dopaminergic because they do not release dopamine and lack other molecular components required for dopamine synthesis, such as L-amino acid decarboxylase, dopamine transporter and vesicular monoamine transporter-2 (Xenias et al., 2015). Third, it is unclear whether FANS are projection neurons or interneurons. For the 10 FANS we successfully labeled with neurobiotin (Figure 12), axonal arborizations were visible and observed to ramify locally; they were never observed to send long-distance projections beyond their dendritic fields (see Figures 12D,E). This observation suggests that FANS, like THIN (Ibáñez-Sandoval et al., 2010), are likely to be interneurons.

## Conclusions

We have discovered FANS, a novel type of neuron in the mouse dorsal striatum. The unique electrical and morphological properties of these neurons add a novel cellular element to striatal local circuitry; future work will be needed to determine the functions of these cells.

## Ethics Statement

All mice used in the experiments were 6–14 weeks old of either sex and all procedures were approved by the Institutional Animal Care and Use Committee of Biopolis, Agency of Science and Technology, Singapore.

## Author Contributions

MM and GA designed the experiments. MM performed the experiments. MM and AN analyzed the data. MM, AN and GA wrote the article.

## Conflict of Interest Statement

The authors declare that the research was conducted in the absence of any commercial or financial relationships that could be construed as a potential conflict of interest.
